# Characterization of an Acute Muscle Contraction Model Using Cultured C2C12 Myotubes

**DOI:** 10.1371/journal.pone.0052592

**Published:** 2012-12-31

**Authors:** Yasuko Manabe, Shouta Miyatake, Mayumi Takagi, Mio Nakamura, Ai Okeda, Taemi Nakano, Michael F. Hirshman, Laurie J. Goodyear, Nobuharu L. Fujii

**Affiliations:** 1 Department of Health Promotion Sciences, Graduate School of Human Health Sciences, Tokyo Metropolitan University, Tokyo, Japan; 2 Research Division, Joslin Diabetes Center and Department of Medicine, Harvard Medical School, Boston, Massachusetts, United States of America; University of Warwick – Medical School, United Kingdom

## Abstract

A cultured C2C12 myotube contraction system was examined for application as a model for acute contraction-induced phenotypes of skeletal muscle. C2C12 myotubes seeded into 4-well rectangular plates were placed in a contraction system equipped with a carbon electrode at each end. The myotubes were stimulated with electric pulses of 50 V at 1 Hz for 3 ms at 997-ms intervals. Approximately 80% of the myotubes were observed to contract microscopically, and the contractions lasted for at least 3 h with electrical stimulation. Calcium ion (Ca^2+^) transient evoked by the electric pulses was detected fluorescently with Fluo-8. Phosphorylation of protein kinase B/Akt (Akt), 5′ AMP-activated protein kinase (AMPK), p38 mitogen-activated protein kinase (p38), and c-Jun NH2-terminal kinase (JNK)1/2, which are intracellular signaling proteins typically activated in exercised/contracted skeletal muscle, was observed in the electrically stimulated C2C12 myotubes. The contractions induced by the electric pulses increased glucose uptake and depleted glycogen in the C2C12 myotubes. C2C12 myotubes that differentiated after exogenous gene transfection by a lipofection or an electroporation method retained their normal contractile ability by electrical stimulation. These findings show that our C2C12 cell contraction system reproduces the muscle phenotypes that arise *in* *vivo* (exercise), *in situ* (hindlimb muscles in an anesthetized animal), and *in* *vitro* (dissected muscle tissues in incubation buffer) by acute muscle contraction, demonstrating that the system is applicable for the analysis of intracellular events evoked by acute muscle contraction.

## Introduction

Exercise induces various types of metabolic event in skeletal muscles, such as increased glucose uptake and fatty acid oxidation. Recently, researchers have turned their attention to the intracellular mechanisms that elicit exercise-induced metabolic events. The rat-derived muscle cell line L6 and mouse-derived muscle cell line C2C12 are frequently used to study intracellular signaling in skeletal muscle cells. The advantages of these cell lines include reducing the use of animals, easy transfection of exogenous DNA or siRNA, a shorter experimental period, homogeneous and easy to culture as clone cells, and avoidance of the influence of systemic factors from other organs. However, a deficiency in contractibility is a major experimental shortcoming of these muscle cell lines. It has recently been reported that cultured rat muscle on silicon cantilevers [1] or C2C12 cells on collagen film [2] can be successfully contracted using electric pulses generated by a microelectromechanical system or an artificial muscle actuator. However, these systems require special devices and are not easy to apply to biochemical studies. More recently, Nikolic et al. reported an acute and chronic exercise model using human cultured myotubes stimulated electrically [3]. Their system is useful as an *in* *vivo* muscle contraction model, although it requires a muscle biopsy from human subjects, obtained after approval from an ethics committee, and the isolation of satellite cells by a specialized technique. In commercially available cells, Kanzaki’s group reported a new contraction system, in which C2C12 myotubes were cultured in conventional culture dishes and electrically stimulated with commercially available carbon electrodes [4], [5]. This system is easy to manipulate and is useful for analyzing the biochemical and molecular biological phenomena induced by muscle contraction. However, this system requires 24 h of pre-stimulation with low-voltage electric pulses for myotube contraction. Since control myotubes are also required to be placed under pre-stimulation conditions before the main contraction session, any changes that override pre-stimulation in the main contraction session must be observed and counted as a contraction-induced effect. Therefore, primary contraction without pre-stimulation would be more suitable for detecting contraction-induced effects in C2C12 cells.

We have developed a new muscle contraction system using C2C12 myotubes without any pre-stimulation. The goal of this study was to determine whether our C2C12 contraction system is an appropriate model for analyzing signal transduction induced by acute contraction. To resolve this issue, we evaluated intracellular calcium ion (Ca^2+^) transient, phosphorylation of intracellular marker proteins, and glucose metabolism profiles. These contraction-induced biological phenomena are typically observed in established muscle contraction systems, such as *in* *vivo* treadmill or wheel cage running, electrically evoked *in situ* contraction in anesthetized animals, and contraction of isolated muscles incubated *in* *vitro* [6]–[8]. The current study clearly shows that the C2C12 cell system reproduces these muscle phenotypes, demonstrating that this is a suitable model of muscle contraction applicable for studies of muscle cell biology and physiology in acute contraction.

## Materials and Methods

### Cell Culture

C2C12 myoblasts (American Type Culture Collection, Manassas, VA, USA) were seeded into 4-well rectangular plates, the surface of which was coated with Matrigel (Becton, Dickinson and Co., Franklin Lakes, NJ, USA), at a density of 2.5×10^5^ cells/well, with 3 mL of growth medium comprising Dulbecco’s Modified Eagle’s Medium (DMEM; 25 mM glucose; Invitrogen, Carlsbad, CA, USA) supplemented with 10% fetal bovine serum (Bio West, Nuaillé, France) and 1% penicillin-streptomycin. This was maintained in an incubator at 37°C under a 5% CO_2_ atmosphere. On reaching confluence, the medium was switched to a differentiation medium consisting of DMEM supplemented with 2% calf serum (Bio West) and 1% non-essential amino acids (Invitrogen) (day 0). Five days after differentiation, cells were used for contraction study.

### Cell Contraction System

**Figure 1:**
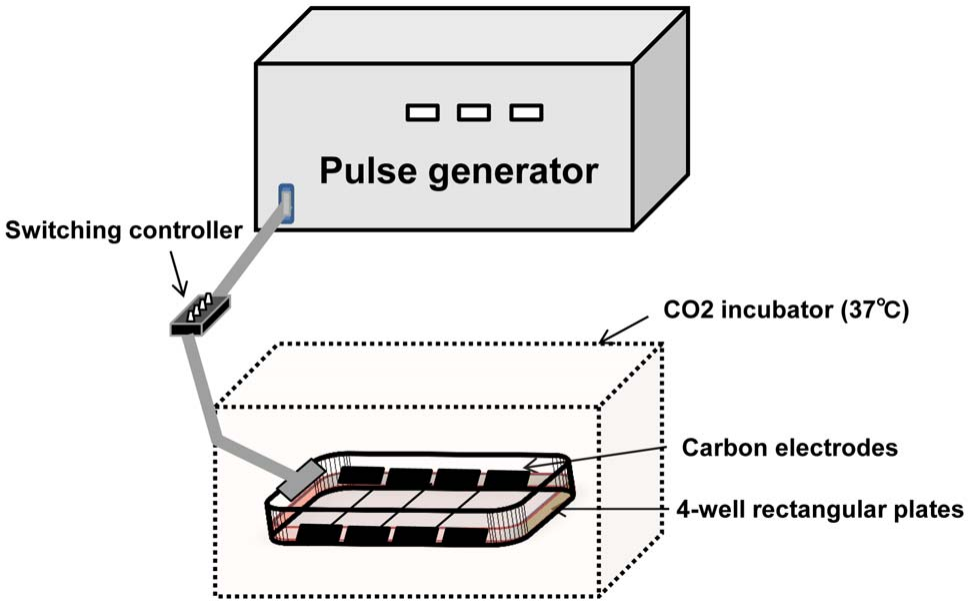
Experimental set-up for C2C12 myotube contraction system. A 4-well or 8-well plate was connected to an electrical stimulation apparatus that had a pair of carbon electrodes for each well. Two wells were for control (no contraction) and the other two were for electrical stimulation in order to induce C2C12 myotube contraction. On-off control was regulated by a switching controller placed between the electrical pulse generator and the apparatus. C2C12 myotubes were stimulated with electric pulses of 50 V at 1 Hz for 3 ms at 997-ms intervals for a given time period in an incubator at 37°C.

The medium was changed to 3 mL of fresh Krebs-Ringer buffer (KRB) immediately before the contraction study. The 4-well plates were connected to the electrical stimulation apparatus, a 4-well or 8-well C-Dish (Ion Optix Corp., Milton, MA, USA), and stimulated by electric pulses generated by an electrical pulse generator (Uchida Denshi, Hachioji, Japan). Differentiated C2C12 myotubes were stimulated with electric pulses of 50 V at 1 Hz for 3 ms at 997-ms intervals for a given time period in an incubator at 37°C (Fig. 1). The contractile activity of the cells was evaluated as the distance shortened between specified two points on a myotube using a motion analyzer (VW-9000; Keyence, Osaka, Japan). Change in the distance was tracked during contraction. The change in the distance at 0, 1, 2, or 3 h after the onset of stimulation is expressed as the integral value of the area under the curve for 5 s (the value is converted to positive number). After contraction, cells were harvested with 300 µL of ice-cold lysis buffer containing 50 mM Tris-HCl (pH 7.5), 10 mM beta-glycerophosphate, 5 mM sodium pyrophosphate tetrabasic, 1 mM sodium orthovanadate, 1 mM ethylenediaminetetraacetic acid (pH 8.0), 1% Nonidet P-40, 150 mM sodium chloride, 10 mM sodium fluoride, 10 mg/L leupeptin, 3 mM benzamidine, 5 µg/mL aprotinin, and 1 mM phenylmethylsulfonyl fluoride. Harvested cells were sonicated and centrifuged at 14,000*g* for 20 min at 4°C, and the supernatant was used for immunoblotting. Protein concentration was determined by the Bradford method.

### Immunoblotting

Cell lysates were separated by sodium dodecyl sulfate-polyacrylamide gel electrophoresis and transferred to polyvinylidene fluoride membranes. The membranes were blocked with Tris-buffered saline comprising 2.5% non-fat dry milk, 2.5% bovine serum albumin, and 0.1% Tween 20. Membranes were incubated overnight with appropriate primary antibodies (Cell Signaling Technology, Boston, MA, USA), and secondary antibody conjugated to horseradish peroxidase (GE Healthcare, Buckinghamshire, UK) was used for detection by enhanced chemiluminescence.

### Intracellular Calcium Ion (Ca^2+^) Concentration Measurements

C2C12 myotubes were washed twice with Phosphate buffered saline (PBS). Ca^2+^ assay buffer, comprising Fluo-8 dye loading solution (AAT Bioquest Inc., Sunnyvale, CA, USA) and DMEM cell medium without serum or antibiotics (1∶1), was added to the wells (2 mL/well) and incubated at 37°C for 30 min. Myotubes were stimulated with electric pulses of 50 V at 1 Hz for 3 ms at 997-ms intervals and observed under a fluorescence microscope (Keyence) at excitation and emission wavelengths of 480 and 510 nm, respectively. The fluorescence intensity was analyzed with a BZ-II analyzer (Keyence) at five arbitrary points.

### Transfection

C2C12 myoblasts at 50–60% confluence were used for transfection. C2C12 myoblasts were transiently transfected with 2 µg of pCAGGS-wild-type c-Jun NH2-terminal kinase 1 (JNK1) with N-terminal HA tag (HA-JNK1) [9] or empty pCAGGS as a control with 187.5 µl of Lipofectamine^TM^2000 and 30 µl of Plus reagent (Invitrogen) according to the manufacturer’s instructions. Electroporation was carried out using the Lonza 2D-Nucleofector system (Lonza, Basel, Switzerland). Trypsinized cells were suspended in the growth medium. Then, 6×10^6^ cells were centrifuged (1,000 g×10 min) and the precipitated cells were suspended in 100 µl of nucleofactor V solution and mixed gently with 100 µg of DNA. The mixture was then electroporated (program D-32). Cells were collected and seeded in a plate.

### Glucose Transport

C2C12 myotubes were contracted for 1 h and then used for glucose transport assay. For both stimulation with insulin and contraction, myotubes were contracted in the presence of 20 mU insulin. Glucose transport was assayed as described previously [10], with minor modification. Cells were washed twice with PBS and incubated with 10 µM 2-deoxyglucose comprising 0.5 µCi/mL 2-[^3^H] deoxyglucose and 70 µM mannitol containing 100 µCi/mL [^14^C]-mannitol in KRB at 4°C for 15 min. The reaction was stopped by washing with PBS containing 10 µM cytochalasin B. Cells were harvested in 500 µL of 0.2 M NaOH in PBS. Uptake of 2-[^3^H] deoxyglucose by the cells was determined, and rates of uptake were calculated as previously described [8]. Protein concentration was determined by the Bradford method.

### Glycogen Measurements

Myotubes were harvested with 200 µL of 2 N HCl and sonicated. Fifty microliters of the sample was set aside for protein assay. The remaining 150 µL was hydrolyzed for 2 h at 95°C. The hydrolyzed sample was centrifuged at 13,000 *g* for 20 min, and the supernatant was applied to an Ultrafree-MC10 (Millipore, Bedford, MA, USA) and centrifuged at 13,000 *g* for 10 min to remove the extra protein. The flow-through fraction (50 µL) was neutralized with 10 µL of 2 N NaOH and 50 µL of 3 M Tris-HCl (pH 7.5). Glucose content was measured with a Sigma Hexokinase kit. Glycogen content is expressed as glucose content per µg of protein.

### Lactate Dehydrogenase (LDH) Assay

The concentration of LDH, released from the cytosol of damaged cells into the supernatant, was assayed using an LDH assay kit following the manufacturer’s instructions (Roche, Basel, Switzerland). LDH release (%) was estimated by dividing the amount of LDH in medium by the total amount of LDH in medium and cell lysate [11].

### Statistics

Data are shown as mean ± S.E.M. Unpaired Student’s *t*-test was performed to evaluate statistical differences between the two groups. For multiple comparisons, data were analyzed using one-way ANOVA followed by Student-Newman-Keuls *post hoc* test, and *p*<0.05 was considered to be statistically significant.

## Results

### Cell Contraction

**Figure 2:**
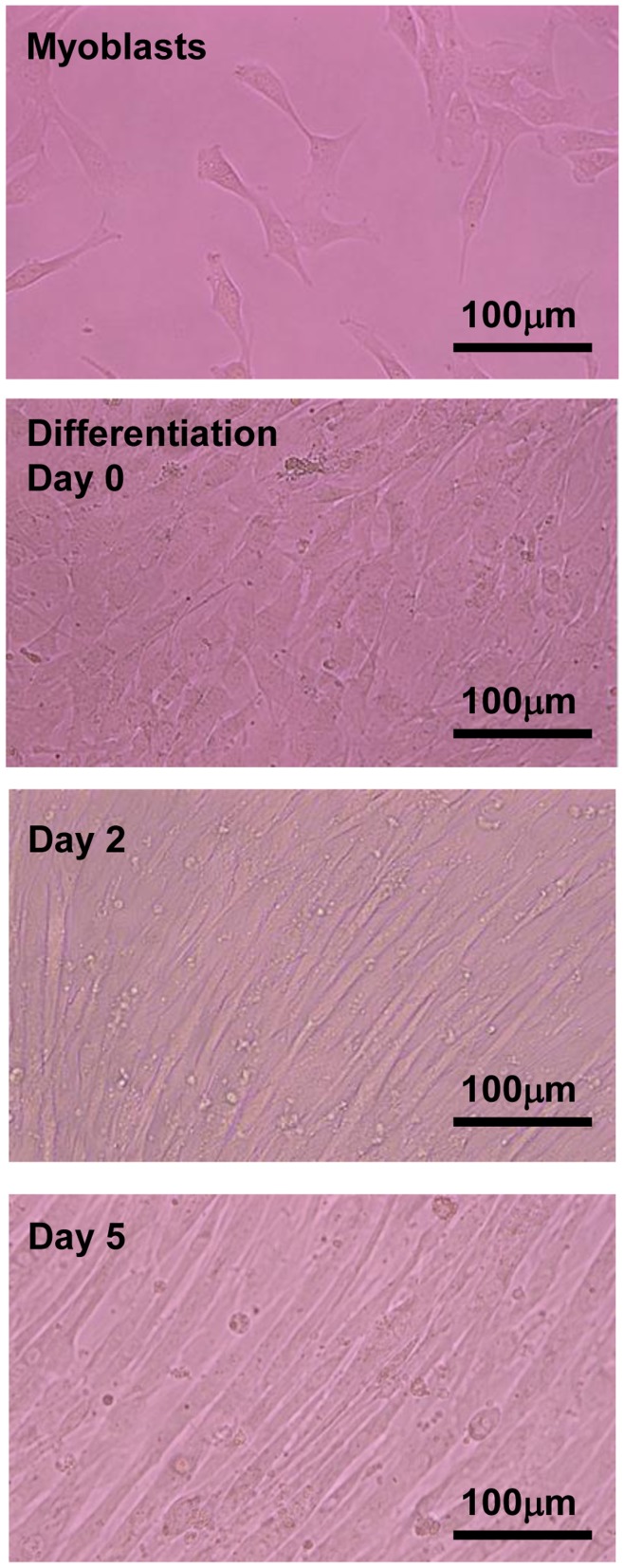
The process of myotube formation in C2C12 cultured cells. The medium was switched to 2% calf serum differentiation medium when the cells reached 90–100% confluence (day 0). Days 2 and 5 indicate the days after switching to differentiation medium. C2C12 myoblasts started to fuse after induction of differentiation, and formed multinucleated myotubes by day 5. All images are shown at 200× magnification.

**Figure 3:**
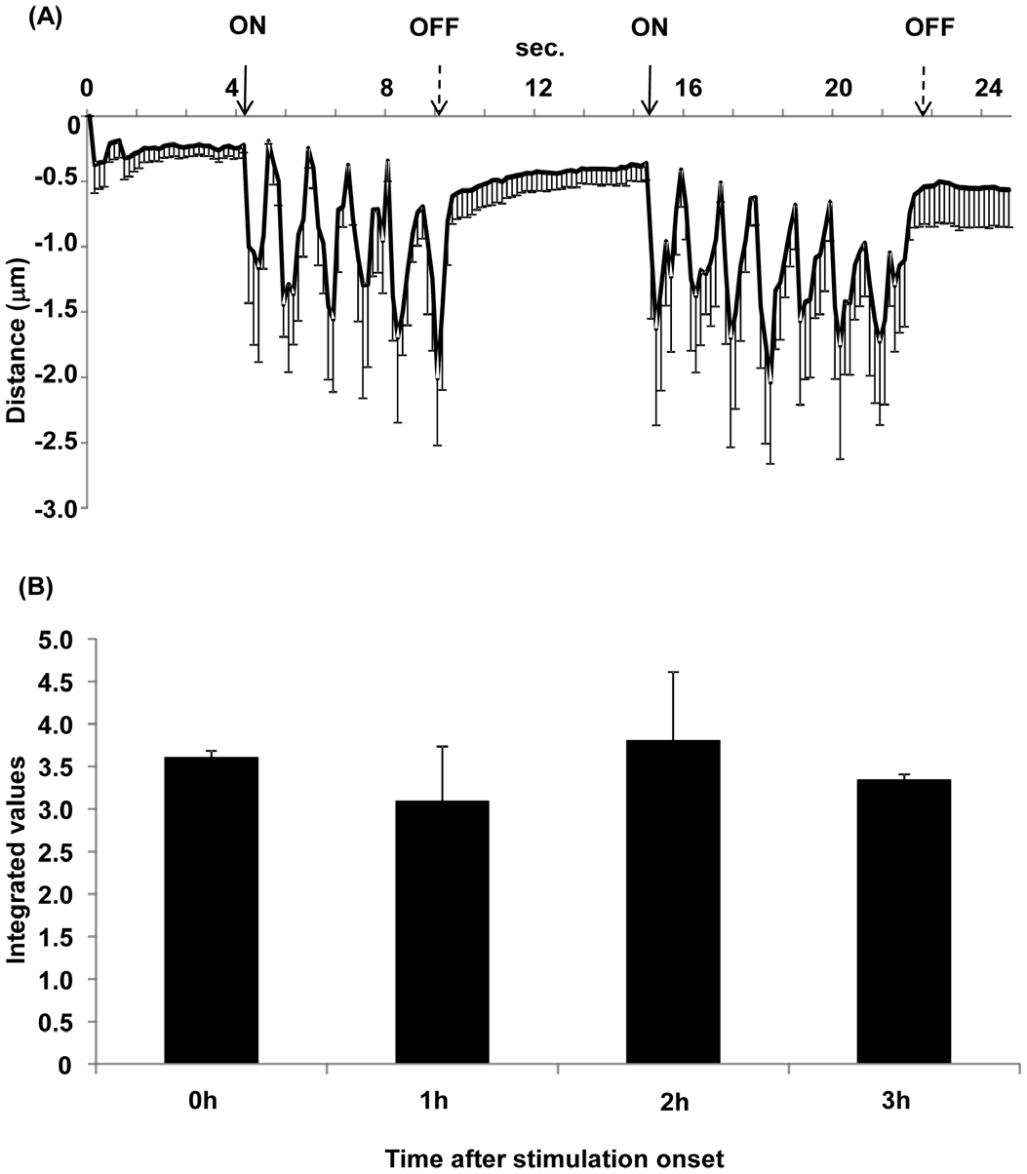
Contractile activity of C2C12 myotubes. (A) The average of contractile activity is shown. The contractile activity of the cells was evaluated as the distance shortened between specified points on a myotube using a motion analyzer. Change in the distance was tracked during contraction. Contraction of the myotubes is synchronized with the intermittent electrical stimulation (see Movie S1). The myotubes were stimulated with electrical pulses of 50 V at 1 Hz for 3 ms at 997-ms intervals. The onset of the electric pulses is shown by solid arrows and their cessation is shown by dashed arrows. Data are mean ± S.E.M., n = 5. (B) Integrated values for 5-s change in the distance at 0, 1, 2, and 3 h after the onset of electrical stimulation. The video is shown in Movies S2, S3, S4 and S5 (0–3 h). The change in the distance at 0, 1, 2, and 3 h after the onset of stimulation are shown as the integral values of the areas above the curves for 5 s (the values are converted to positive number). The integrated values 1, 2, and 3 h after stimulation onset are comparable with that at the onset (0 h). Data are shown as mean ± S.E.M., n = 3.

C2C12 myoblasts were mononuclear in the growth phase (Fig. 2, Myoblasts). When the myoblasts were nearly confluent (Fig. 2, Day 0), the growth medium was switched to a differentiation medium containing 2% calf serum. C2C12 myoblasts began to fuse 2–3 days after the induction of differentiation (Fig. 2, Day 2) and multinucleated myotubes were formed by day 5 (Fig. 2, Day 5). Once the cells had formed multinucleated myotubes on day 5, a few myotubes showed spontaneous contractile activity without any stimulation. When the myotubes were stimulated by electric pulses of 50 V at 1 Hz for 3 ms at 997-ms intervals, most myotubes contracted immediately (Movies S1 and S2). The rhythmic contractile activities were synchronized with the electric pulses. Almost all myotubes in the microscopic field were capable of contraction from the onset to a minimum of 3 h later. To confirm that the contraction was induced by stimulation by the electric pulses, the pulse generator was turned on and off and the contractile activity of the cells was evaluated as distance shortened between specified two points on a myotube using a motion analyzer (Movie S1). As shown in Fig. 3A, contractile activity was synchronized with the electric pulses. Fig. 3B shows the integrated contractile activity for 5 s at 0, 1, 2, and 3 h after the onset of contraction (Movie S2, S3, S4, and S5, respectively). The contractile activities of the myotubes continued consistently for at least 3 h. Myotubes could maintain contraction even for 24 h under these conditions, but some cells were damaged and floated in the medium (data not shown).

**Figure 4:**
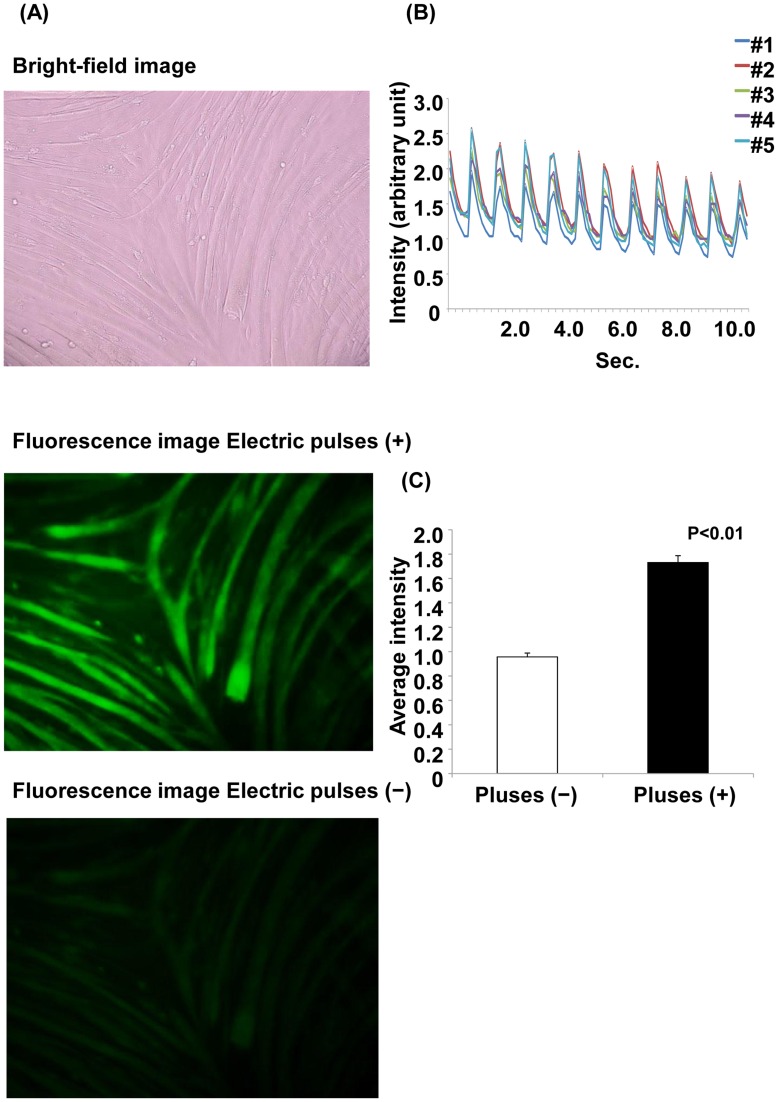
Ca^2+^ fluorescence with and without electrical stimulation. (A) Ca^2+^ fluorescence with and without electrical stimulation. Myotubes were treated with Fluo-8 dye loading solution 30 min before electrical stimulation. The images are shown at 200× magnification. The upper panel shows the bright-field image. The middle panel shows the myotubes with electric pulses, and the lower panel shows the myotubes without electric pulses. (B) Changes in Ca^2+^ fluorescence intensity with electrical stimulation. The fluorescence intensity was analyzed at 5 arbitrary points. Each line shows the raw fluorescence intensity data at each point. (C) The average fluorescence intensity for 11 s is shown. The average fluorescence intensity with electric pulses is significantly higher than that without electric pulses (*p*<0.01, Student’s t-test).

Muscle contraction is regulated by Ca^2+^, which flows into the cytoplasm from the sarcoplasmic reticulum. We evaluated Ca^2+^ transient evoked by electrical pulses using the fluorescent Ca^2+^ indicator Fluo-8. As shown in Fig. 4A, Ca^2+^ concentration was increased by stimulation with electric pulses, and the elevation of Ca^2+^ was synchronized with the electric pulses (Fig. 4B). The average fluorescent intensity with electric pulse stimulation was approximately twice that without the electric pulse (Fig. 4C).

**Figure 5:**
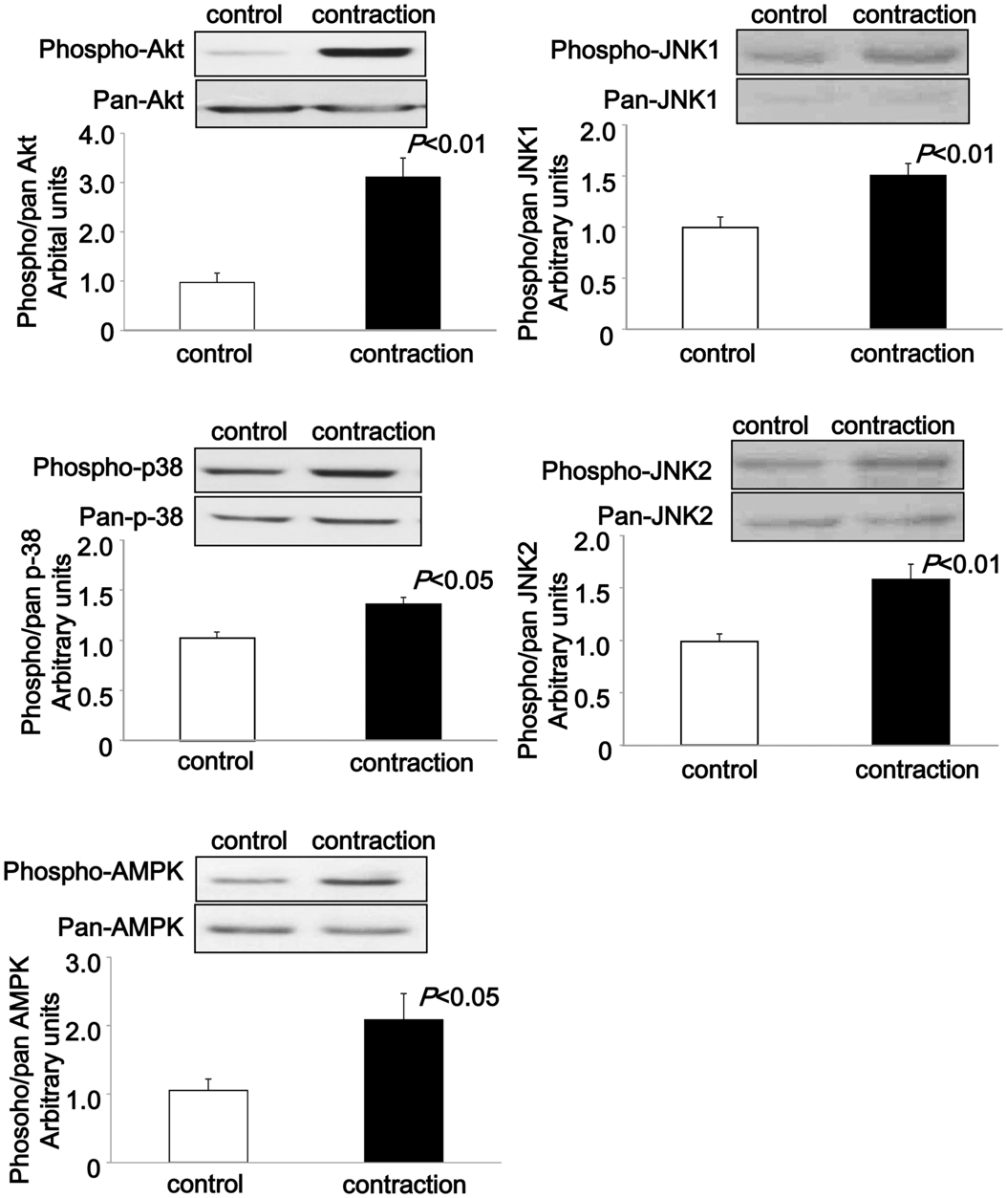
Immunoblotting of phosphoproteins after electrical stimulation for 1 h. C2C12 myotubes were stimulated with electric pulses (50 V, 1 Hz, 3 ms) for 60 min at 37°C. Representative blots of the phosphorylation of Akt (Ser 308), p-38 (Thr180/Tyr182), AMPK (Thr172), and JNK1/2 (Thr183/Tyr185) and their expression levels induced by electrical stimulation for 60 min in C2C12 myotubes are shown. The phosphorylation ratios were calculated by dividing the phosphorylation levels by the protein expression levels. Significant increases in phosphorylated Akt (Ser 308), AMPK (Thr172), p-38 (Thr180/Tyr182), and JNK1/2 (Thr183/Tyr185) were detected after 1 h of contraction. Data are shown as mean ± S.E.M, n = 6–14.

Next, we examined whether some serine/threonine kinases, which are phosphorylated and activated by *in vivo* exercise, as well as *in situ* and *in vitro* muscle contractions [8], [12]–[14], were phosphorylated by contraction of the C2C12 myotubes. The immunoblotting results showed that phosphorylation of protein kinase B/Akt (Akt; Thr308), 5′ AMP-activated protein kinase (AMPK; Thr172), p38 mitogen-activated protein kinase (p38; Thr180/Tyr182), and JNK1/2 (Thr183/Tyr185) was significantly increased by contraction for 1 h (Fig. 5).

Similar increases in phosphorylation of Akt, AMPK, and JNK1/2, but not p-38, were also observed by muscle contraction for 3 h (Fig. S1). These results suggested that biochemical events induced by *in vivo* muscle contraction could be reproduced in our C2C12 contraction system.

### Glucose Uptake and Glycogen Concentration

**Figure 6:**
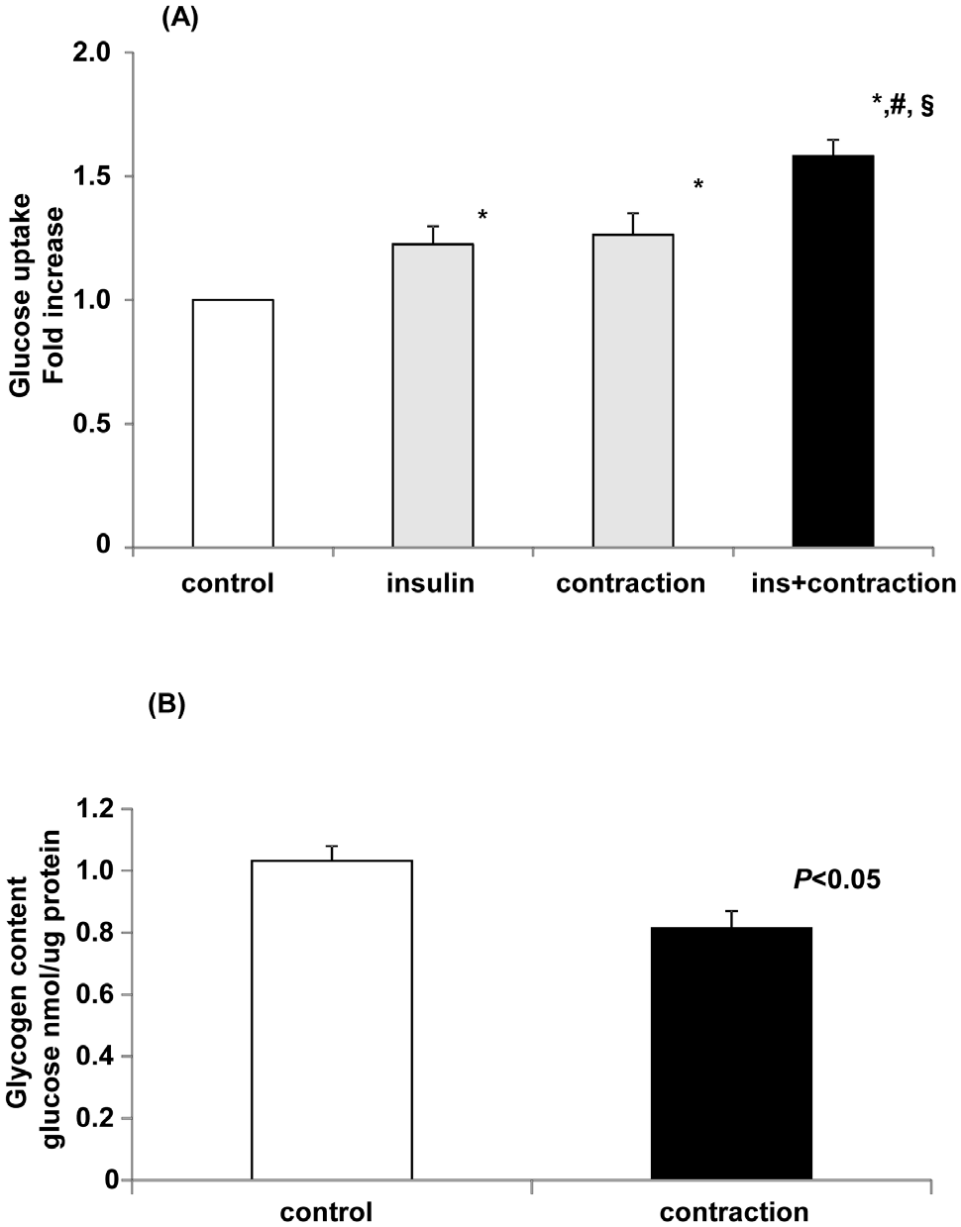
Electrical stimulation induces increased glucose uptake and decreased glycogen content in C2C12 myotubes. (A) Glucose uptake in C2C12 myotubes after contraction. C2C12 myotubes were stimulated by insulin (20 mU/mL), electric pulses, or electric pulses in the presence of insulin in KRB for 1 h, after which 2-deoxy-D-glucose transport was measured. The control was incubated for 1 h at 37°C without stimulation. Data are mean ± S.E.M, n = 8/group. Data were analyzed using one-way ANOVA followed by Student-Newman-Keuls *post hoc* test, with *p*<0.05 considered statistically significant. *significant compared to the control; ^#^significant compared to insulin alone; ^§^significant compared to stimulation alone. (B) Glycogen concentration in C2C12 myotubes after stimulation by electric pulses. C2C12 myotubes were stimulated by electric pulses (50 V, 1 Hz, 3 ms) for 1 h at 37°C. Glycogen concentration is shown as the glucose concentration per µg of protein. The glycogen concentration in contracted C2C12 myotubes was significantly reduced. Data are shown as mean ± S.E.M., n = 5–7.

Exercise or muscle contraction increases glucose uptake in skeletal muscle by a mechanism that is independent of the insulin-signaling pathway [15]. In our C2C12 system, glucose uptake was significantly increased by insulin or muscle contraction by 23% or 26%, respectively (Fig. 6A). When myotubes were stimulated by both insulin and contraction simultaneously, glucose uptake increased additively by 58%. This type of additive effect is a discriminative property of skeletal muscle and is known to result from the different regulatory pathways of insulin- and muscle contraction-induced glucose uptake [8], [16]. Glycogen in skeletal muscle is the primary source of energy during muscle contraction. Therefore, we measured whether contraction for 1 h reduces glycogen levels in C2C12 myotubes. As shown in Fig. 6B, the glycogen level in contracted C2C12 myotubes was significantly lower than that in control C2C12 myotubes, which is in agreement with the *in vivo* exercise model [17]. These results suggested that our C2C12 system is suitable for analyzing changes in glucose metabolism during muscle contraction.

### Transfection of a Foreign Gene

**Figure 7:**
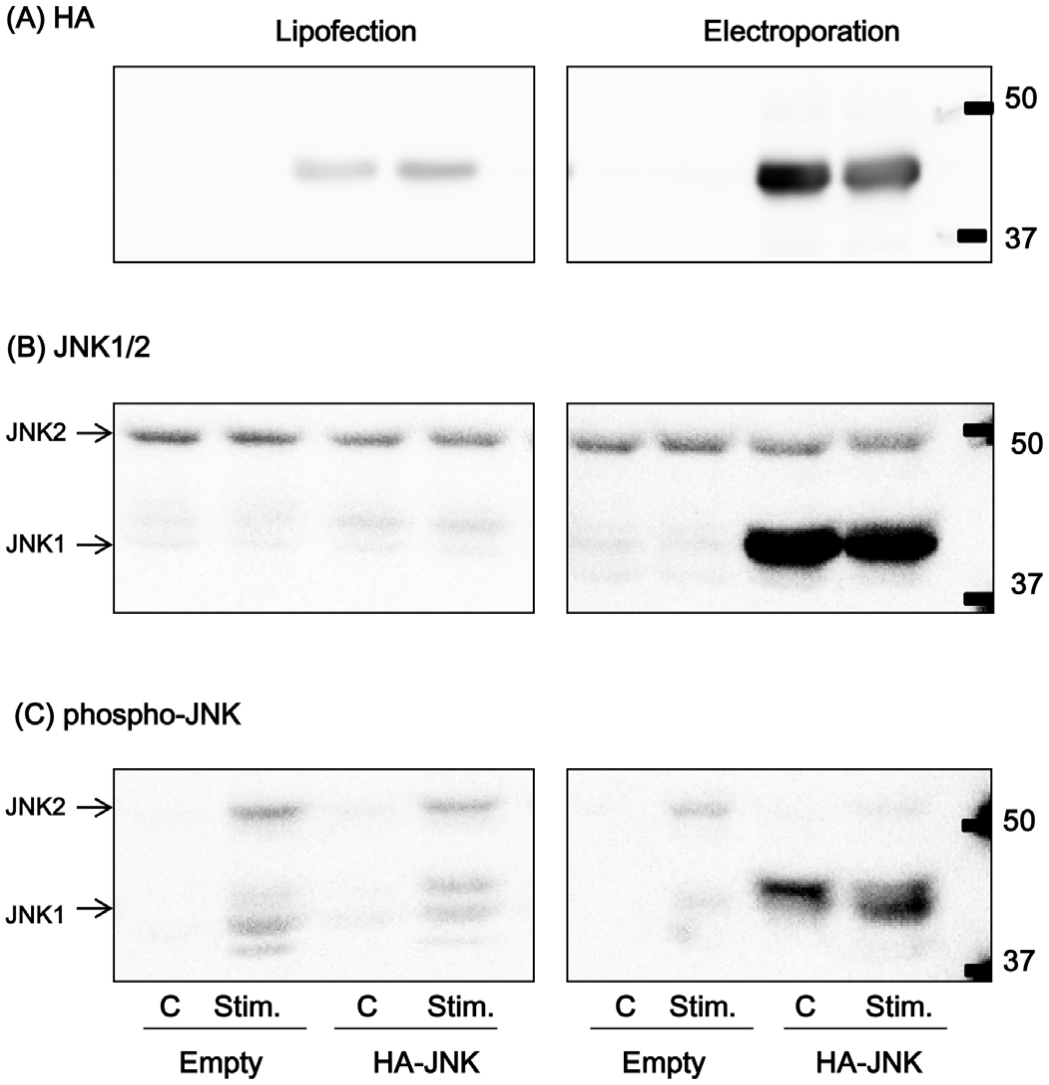
Expression and phosphorylation of JNK1 in C2C12 myotubes. JNK1 with N-terminal HA tag (HA-JNK1) or empty vector was transfected by either a lipofection method or an electroporation method. Detection was carried out using HA-HRP antibody (A), JNK1/2 antibody (B), and phospho-JNK antibody (C). “C” indicates control samples without any stimulation; “Stim.” indicates contracted samples stimulated by electric pulses (50 V, 1 Hz, 3 ms) for 1 h.

To evaluate whether the normal muscle contractile ability of C2C12 myotubes is maintained after transfection of a foreign gene, JNK1 expression vector was transfected into C2C12 myoblasts, which then differentiated into myotubes. JNK1 protein was successfully expressed, by both a lipofection method and an electroporation method, at approximately 3- and 30-fold over the control level, respectively (Fig. 7). The electroporation method seemed to be more suitable than the lipofection method, considering the respective exogenous JNK1 expression levels (10-fold higher in the electroporation method). The muscle contractile ability of JNK1-transfected C2C12 myotubes evaluated using a motion analyzer was comparable to those of both non-transfected myotubes and empty vector-transfected myotubes (data not shown). The immunoblotting result showed that contraction-induced phosphorylation of JNK1 was increased, as expected (Fig. 7).

### LDH Activity

**Figure 8:**
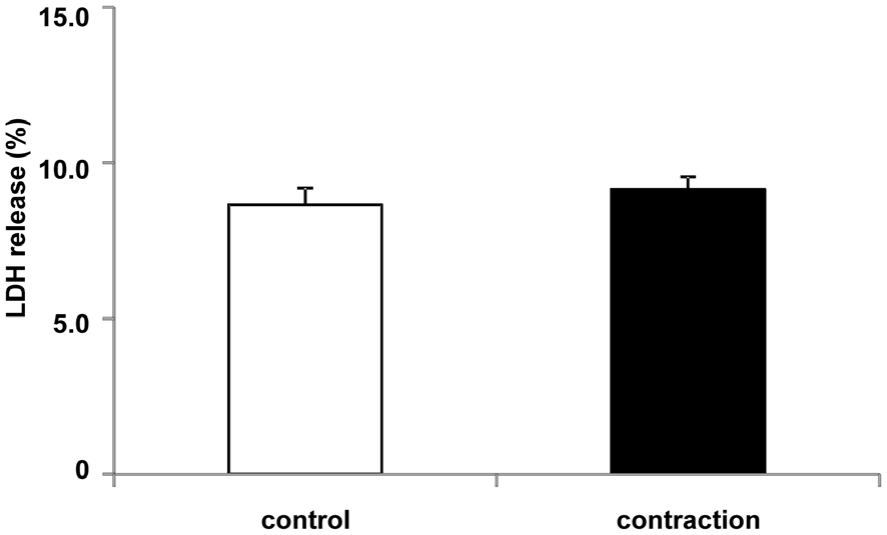
LDH activity in the culture medium after 1 h of contraction in C2C12 myotubes. C2C12 myotubes were stimulated by electric pulses (50 V, 1 Hz, 3 ms) for 1 hr. There was no significant difference between non-contracted control and the contraction group (n = 6). LDH release (%) was calculated by dividing the amount of LDH in medium by the total amount of LDH in the medium and lysate (Materials and Methods).

To confirm that C2C12 myotubes were not damaged during contraction, LDH was measured in the culture medium after 1 h of contraction. LDH is present in the cytoplasm of intact cells and is rapidly released into the culture medium after cell damage. The LDH concentration in the medium of C2C12 myotubes stimulated by electric pulses of 50 V at 1 Hz for 3 ms at 997-ms intervals for 1 h was unchanged from that in the control cell culture medium, suggesting that the cells were not damaged by contractile conditions (Fig. 8).

## Discussion

To determine whether our muscle contraction system with C2C12 myotubes is suitable as a new model for analyzing acute contraction-induced biological events, we attempted to reproduce the cellular events that have been well established to occur in *in vivo* exercise, as well as *in situ* and *in vitro* muscle contractions in human and animals [6]–[8]. We confirmed the insulin- and contraction-induced glucose transport, Ca^2+^ influx, and phosphorylation of several kinases that have typically been characterized in animal exercise models or *in vitro* models. In addition, the contractile ability of C2C12 myotubes was found not to change upon the transfection of foreign genes and the expression of corresponding proteins, demonstrating that our C2C12 model is suitable for defining the physiological role of intracellular signaling evoked by muscle contraction, which is the major function of this tissue. A major advantage of the system is that contraction can be achieved from the onset of electrical stimulation without any pre-stimulation phase, and the contraction activity is maintained at a constant level for at least 3 h. Therefore, it is possible to use non-contracted C2C12 myotube sets as controls for comparison with the biological events evoked in contracted myotubes.

Attempts to construct similar systems using cultured myotubes that mimic muscle contraction have been previously reported. For example, some researchers have proposed a cell stretching system using flexible-bottomed wells [18], [19]. Using this system, they observed Ca^2+^ release into the intracellular spaces of myotubes. Although this stretching system has recently been widely accepted as a model for analyzing muscle biology, passive stretching does not always induce cellular responses that are identical to those of active or forced contraction. For example, passive stretching of isolated mouse skeletal muscle *in vitro* does not increase AMPK activity, which is a representative response to active or forced muscle contraction *in vitro* and *in vivo* [20].

There are a few reports of active contraction of myotubes differentiated on a collagen or fibrin gel on glass slides and stimulated electrically with custom-made electrical devices [2], [18], [21]. These systems have recently become the focus of artificial muscle development. However, these systems require a specific device and are not easy to manipulate. In contrast, Fujita et al. applied a commercially available device that is typically used for cultured cardiomyocytes in order to maintain the autonomic contractility of the cells. After eight days of differentiation, C2C12 myotubes were pre-stimulated with this device with electric pulses of 40 V/60 mm for 24 ms at 1 Hz for 2 h or 0.5 Hz for 9 h to promote *de novo* sarcomere synthesis and assembly [4]. This process induced vigorous contractile activity in C2C12 myotubes under the same contractile conditions as pre-stimulation. Subsequently, the researchers improved their system by differentiating myotubes for six days and pre-stimulating them under low electrical pulse conditions (40 V/60 mm, 1 Hz, 2 ms) for 24 h, followed by primary stimulation under the same contractile conditions as pre-stimulation [10]. It was noted that an extremely low frequency of electrical stimulation (0.1 Hz) was necessary during the resting phase after pre-stimulation to obtain strong contractile activity with the primary stimulation [10]. In our study, C2C12 myotubes contracted at the onset of electrical stimulation and the contractile activity was sustained for a minimum of 3 h. Another advantage of our system is that control cells do not require pre-stimulation treatment, which results in the maintenance of their basal state. Therefore, this system can provide higher sensitivity for detecting muscle contraction effects and can eliminate differences between experimental and control groups that may result from different cell culture conditions. In our study, high-glucose DMEM medium was used as both growth and differentiation medium, although non-essential amino acids was added to the differentiation medium. Furthermore, differentiation was induced when myoblasts reached confluence, which provides thicker myotubes with dense agglomerated nuclei. Under these conditions, C2C12 myotubes are well differentiated and form contractile myotubes until day 5.

More recently, Nikolic et al. reported that primary cultured myotubes originally obtained from human muscle tissue were electrically contracted for 1, 24, or 48 h and showed increased glucose uptake and oleic acid uptake, suggesting that this model can be used as an acute and a chronic exercise model [3]. Since primary cells obtained from a subject’s tissue generally retain their *in vivo* characteristics, their model is also useful for the analysis of contraction-induced muscle phenotypes. On the other hand, it has been pointed out that primary cells are heterogeneous, which can cause considerable variation in the experimental results, require a cumbersome procedure (muscle biopsy, elimination of other types of cell, such as fibroblasts) for the preparation of cells, and convey a risk of viral contamination of viruses originating from the subject. Therefore, the choice of cell type, primary cell or clone (C2C12 cell) cell, should be based on the specific purpose of the study. It should be noted that the use of both of these cell types can compensate for any of their individual weaknesses.

We tested a variety of electrodes for the electrical stimulation, including copper, brass, and platinum. However, efficient contraction was not obtained with these electrodes and the contraction did not last for hours (data not shown). In addition, we failed to achieve contraction with L6 rat myotubes. Differentiation and stimulation conditions for the contraction of these myotubes should be carefully investigated in the future.

In conclusion, the C2C12 contraction system is a useful tool for analyzing biological events in skeletal muscle cells and can be a useful model for assessing the effects of acute muscle contraction. Using this system, it is possible to recreate Ca^2+^ transient, phosphorylation of several serine/threonine kinases, and changes in glucose metabolism, all of which are typically observed in existing contraction systems including physical exercise. In addition, the contractile ability of C2C12 myotubes does not change upon the transfection of foreign genes and the expression of their corresponding proteins, demonstrating that our C2C12 model is suitable for defining the physiological role of intracellular signaling evoked by muscle contraction, which is the major function of this tissue.

## Supporting Information

Figure S1 Immunoblotting of phosphoproteins after electrical stimulation for 3 h. C2C12 myotubes were stimulated with electric pulses (50 V, 1 Hz, 3 ms) for 3 h at 37°C. Representative blots of the phosphorylation of Akt (Ser 308), p-38 (Thr180/Tyr182), AMPK (Thr172), and JNK1/2 (Thr183/Tyr185) are shown. The phosphorylation ratios were calculated by dividing the phosphorylation levels by the protein expression levels. Significant increases in phosphorylation of Akt, AMPK, and JNK1/2, but not p-38, were observed. Data are shown as mean ± S.E.M, n = 8.
(TIF)

Movie S1 The rhythmic contractile activities were synchronized with the electric pulses in C2C12 myotubes. C2C12 myotubes contracted immediately after stimulation with electric pulses of 50 V at 1 Hz for 3 ms at 997-ms intervals. Contractile activity was shown in Fig. 3A.
(ZIP)

Movie S2 Contracting C2C12 myotubes at 0 h after the onset of stimulation with electric pulses of 50 V at 1 Hz for 3 ms at 997-ms intervals.
(ZIP)

Movie S3 Contracting C2C12 myotubes at 1 h after the onset of stimulation with electric pulses of 50 V at 1 Hz for 3 ms at 997-ms intervals.
(ZIP)

Movie S4 Contracting C2C12 myotubes at 2 h after the onset of stimulation with electric pulses of 50 V at 1 Hz for 3 ms at 997-ms intervals.
(ZIP)

Movie S5 Contracting C2C12 myotubes at 3 h after the onset of stimulation with electric pulses of 50 V at 1 Hz for 3 ms at 997-ms intervals.
(ZIP)
