# Supplementary figures and images for: Characterization of an Acute Muscle Contraction Model Using Cultured C2C12 Myotubes
Source: PLoS One. 2012 Dec 31;7(12):e52592. doi: 10.1371/journal.pone.0052592 (PMC3534077; doi:10.1371/journal.pone.0052592)

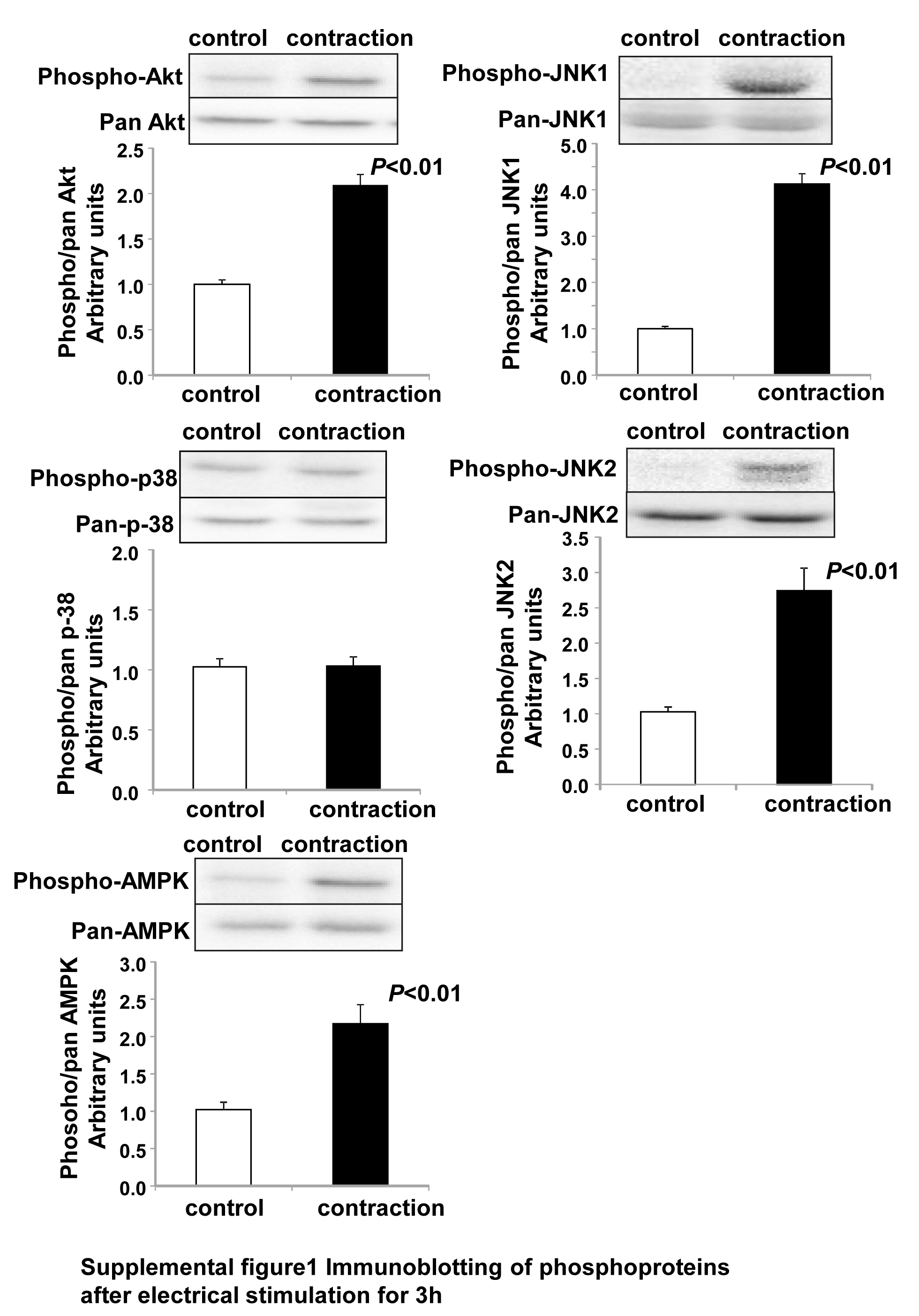

Supplement: Figure S1 — Immunoblotting of phosphoproteins after electrical stimulation for 3 h. C2C12 myotubes were stimulated with electric pulses (50 V, 1 Hz, 3 ms) for 3 h at 37°C. Representative blots of the phosphorylation of Akt (Ser 308), p-38 (Thr180/Tyr182), AMPK (Thr172), and JNK1/2 (Thr183/Tyr185) are shown. The phosphorylation ratios were calculated by dividing the phosphorylation levels by the protein expression levels. Significant increases in phosphorylation of Akt, AMPK, and JNK1/2, but not p-38, were observed. Data are shown as mean ± S.E.M, n = 8. (TIF) [file pone.0052592.s001.tif]
